# Operative Dentistry

**Published:** 1882-06

**Authors:** Geo. S. Miles

**Affiliations:** Jerseyville.


### ARTICLE III.

### *Operative Dentistry.*

BY DR. GEO. S. MILES, OF JERSEYVILLE.

[Read before the Illinois State Dental Society, held at Rock Island, May 10, 1881.]

In responding to the request of the Executive Committee, I do not expect to say anything that will be new to the members of this Society.

I presume the committe felt that this branch of the profession was of such importance that some space in the programme should be allotted to it, if but to open the discussion, which may be so profitably continued by the members here present.

Nearly all the different pursuits in life, trades and professions, are divided into sections or branches in such a manner that a person following one or two will achieve greater perfection and influence than if he endeavored to attend to all the different branches of the trade or profession with which he is connected.

In the manufacture of the watch; an article so indispensable to every gentleman; instead of the manufacture of all its many parts being confided to one individual, the mechanism of it is divided into many sections, and one person gives his or her attention to one particular part, another to another, and so on until the beautiful chronometer is produced, each part fitting with exact nicety, and keeping time to the delight and admiration of the most critical.

The steam engine, printing press, electric, telegraphic machine, and many other important and wonderful invention that are interwoven with the welfare and business existence of the race, could never have been produced and made to perform the duties required of them with that exact nicety and satisfaction but for the fact that their several parts were allotted each to skilled workmen of tried capacity.

The same principles applies to the several professions that does no•mechanical arts. The practitioner at law could not have attained the high position that many of the profession in this country now occupy, had they undertaken to counsel and advise within the whole range of jurisprudence, for it is patent to all that the most eminent practitioners, those who have accomplished the most for their clients, as well as themselves, are those who have confined themselves to a limited range of practice.

The same principle applies to the practice of medicine, and especially is such the case if dentistry is a specialty in medicine. Not many years ago it was not unusual for a person to combine the practice of dentistry with some other calling or profession. It seemed to be quite popular for clergymen to divide their clerical labor with dental practice; I suppose, with the idea that while ministering to their spiritual needs they could also attend to their physical requirements, and at the same time replenish their exchequer. So far as my knowledge extends, these gentlemen have been about equally successful as preachers and dentists, and as the standard of dentistry has advanced, they have found there was enough range in their profession to engage one person's attention, and they have withdrawn, and many are now in the insurance business, for which I presume they are better adapted. All agree at this day that there is enough in the practice of dentistry to demand a person's whole attention. How best to acquire the proper information and practical experience to fit a person for the work, is the conundrum. A thorough knowledge of medicine is no disadvantage to the dentist, but it is certainly an accomplishment. The same may be said of law and theology.

It is not deemed important that a practitioner at law should understand theology, or that the teacher of theology should be profound in the mysteries of law; and while dentistry may be considered to be more intimately connected with medicine than the other professions are with each other, the graduate of medicine is very poorly fitted to practice dentistry; and yet he should be, and is, I doubt not, prepared to practice surgery, treat diseases of the eye and ear, and many other diseases of the system which are recognized as specialties in medicine when practiced as such. Wherever dentistry is introduced in a medical school an additional corps of teachers is required, and at the present day, as they exist, I have no doubt the graduate of medicine obtains information that he would not receive but for this fact. At the same time the dental student is benefited from what instruction he may receive in medicine. But as no one person can know everything, it does not seem important that a dental student should be required to pursue and understand all of those branches of medicine and the nature and treatment of all the diseases of the human system, when he never expects to make any use of the instruction thus obtained; but thorough instruction in all those branches of dental science and art, the pathology of the dental organs, and the treatment of those diseases in any way connected with the oral cavity, or appertaining to the duties of the dental practitioner, would prepare the student better for the work selected. The mind of the dental student should not be diverted from his subject, but his whole attention given to the central aim of his life.

The subject of operative dentistry presents a wide field for scientific investigation and artistic study. From infancy to old age the operator is called upon to diagnose the many troubles of the oral cavity, and exercise what knowledge and skill he possesses in ministering to the needs of those thus afflicted. Very few people in this era are exempt from the necessity for the services of a dentist; hence, it is very important that thorough investigation be made of each case that presents itself so that correct conclusions may be drawn, and a proper course of treatment decided upon.

In examining the teeth, a delicate instrument is needed to inspect carefully each tooth, the interstices in the grinding surfaces, between the teeth, around the necks of the teeth, and often under the gums. In many cases this can only be done satisfactorily by the use of floss silk, drawn between the teeth, and by wedging the teeth apart.

Before commencing to fill teeth that are decayed, it is often best to remove every particle of tartar that may be attached to them. In some cases it is imperative. No one should allow a patient to go from his office understanding her work completed, who desires her teeth put in proper condition, without seeing they are perfectly free from tartar, and the patient instructed how to keep them free from the accumulation.

Upon examination of a case we find quite a number of teeth decayed in different parts of the mouth- In filling teeth that are decayed upon the proximal surfaces of the incisors, sufficient space should be made to prepare the cavity, insert the filling, condense and finish the same in a proper manner. How best to obtain this space so that the filling may be firmly condensed around the margins, and after completion the tooth present the proper shape for durability and general appearance, is the question.

Judgment must be used as the case presents itself, depending upon the quality, shape and position of the teeth and the extent of the decay. Where the incisors are considerably wider at and near the cutting edge, the proximal surfaces touching each other, with decay near the neck of the tooth, reaching near the point of contact, I think the space should be obtained by wedging. This may be accomplished with cotton or orange wood, as you prefer. Very little, if any, filling should be done, from the fact that very soon after the operation is completed, the teeth will come in contact again, and the surfaces filed would be broader, and without the rounded contour which was natural to them. Substances would collect between them, and decay would be more liable to supervene. Where the decay is near the cutting edge, in proximal cavities, and the position of the teeth to each other such that they would impinge upon each other even after considerable tooth structure was removed, I would prefer not to injure the shape of the tooth by removing much of the tooth substance, but obtain space by wedging and by taking away only the slender edge of the walls, or portion of the walls, and retain the proper shape of the teeth—the contour of the filling depending upon the extent of the decay, but so shaped that as small a portion as possible would press upon the adjacent tooth. A flat surface should be avoided in proximal fillings, and care should be taken that the other extreme be not reached, and a too prominent contour given to the filling for permanency.

If practicable, the teeth should be left, after the fillings are completed, with a narrow space betweeen them, in order that they may be the more easily kept clean. The molars and bicuspids not being so exposed to view, while it is desirable to retain a proper shape, I do not deem it as important to retain so closely the shape of the tooth as in the incisors, but such separations should be made and frail walls cut away as will enable the operator to insert a filling for the longest period.

Much has been said and written during the past few years in regard to materials for filling teeth—considerable that is quite valuable ; not a little that I believe had better not have been written, if indeed it has not been pernicious in its influence upon others who may have inclined to a system or adopted suggestions without sufficient investigation.

There has been a tendency to run to extremes in the use of materials and appliances in our profession, and also an inclination to follow in grooves or the tracks of others, rather than to exercise one's own judgment of what is best for each individual case.

Of the materials for filling teeth, gold should stand *first* as the *jewel* to take the place of lost tooth structure. By far the larger proportion of teeth may be filled with gold in some of its forms of preparation, in a more durable manner, and present a more beautiful and cleanly appearance, than other now discovered preparations; especially is gold preferable for the incisors and cuspids. There are many large cavities of decay in the molars and bicuspids, where the walls are frail, in which an amalgam preparation may be used, and preserve the teeth for a longer period than if the cavities were filled with gold. Indeed, teeth may be filled with this material the walls of which are so frail that it would be nearly if not quite impossible to fill with gold. Again, the expense of inserting these large gold fillings is such as to debar many from having their teeth attended to, unless some cheaper material can be used. They cannot afford to pay the price for these large gold fillings that would be equitable—at least they think they can not, and they will not—and the question hinges right here: Shall these teeth be allowed to continue in their onward career to destruction, and ultimately be shelled from the mouth as you would shell peas from the pod, by the artificial tooth *fiend*, or shall we arrest the decay and fill these teeth with amalgam or tin, as the case may be? I believe in saving the teeth. One reason for the dislike of amalgam has been, I think, the quality of the preparation, and the neglect in the preparation of the cavity of decay, and the proper introduction and finishing of the filling. Quite as much care should be exercised in filling with amalgam as with gold.

It is equally important to use the rubber dam, for it is very essential to keep all moisture from the filling, while it is being inserted and for an half hour or more after. By this means you are able to finish the filling in a more perfect manner, giving it a finer and more complete finish after the material has hardened considerably, which is a great desideratum.

Attention should be paid to the saving of children's teeth, so that they may be used for mastication, until the natural period for their removal, for the permanent set. Tin foil and gutta-percha are very good materials to use in these teeth, and will generally fulfill the requirements demanded of them.

Many operators have a preference in the amalgams now manufactured. Others seem to act upon the theory that they are all of about the same class, and use the cheapest. Each manufacturer claims his to be equal if not superior to others. The opinions of those qualified to judge, who are not interested in the sale of the article, I think would be preferable. But I think what would be still better would be the appointment of a competent committee by this society, or by the "American Dental Association," to investigate and experiment in regard to the proper ingredients, and the relative proportions of each, until (if possible) they can report a formula of a preparation that will not shrink from the walls, and that is not injurous to tooth structure if proper care is taken in introducing the filling.

The operative dentist is often called upon to perform operations in the oral cavity, other than filling teeth. His ingenuity and skill are not infrequently brought into use in correcting irregularities of teeth. Children's teeth, in consequence of the neglect of parents, or imperfect development of the maxilla, are very often crowded from that symmetrical position designed by nature, and present a very forbidding appearance. In youth this condition of affairs may be changed. The crowded condition being removed, not only is their liability to decay greatly lessened, but a wonderful change wrought in the whole facial expression of the patient. At times this would require the removal of a bicuspid or molar, to make room, but very seldom, if ever, the extraction of any tooth anterior to these; for it is very important that the incisors and cuspids should be saved for general appearance. Various, and in some cases quite ingenious, appliances are constructed for changing the position of the teeth, requiring considerable time in accomplishing the purpose; but often this can be effected very easily by the use of silk ligatures attached to the teeth, connected with rubber rings. Frequently we see an incisor that has been allowed to strike behind the lower teeth. This position may be very readily changed by adjusting a plate over several of the lower teeth, so arranged that the incisors desired to be removed will strike upon an inclined plane in closing the mouth. We see teeth almost every day with exposed pulps. It is highly important to preserve this vital principle of the tooth, if possible. This can be done if there is no inflammation, or chronic disease, in or about the organ, by covering the nerve with Fletcher's or Weston's preparation. The antiseptic pulp dressing of Dr. Spalding I believe is a very good article for this purpose; covering it with oxychloride and, after the zinc filling is hard, fill the tooth as usual. If the pulp is in an inflamed condition or dead, it should be removed to the apex of the root, the nerve cavity purified, and the canals filled, care being taken to hermetically seal the nerve cavity at the apex, that no moisture may permeate the filling

Many teeth are neglected until the crowns are broken off, or nearly so. In many of these cases, if the root is strong, I think it better to attach an artificial crown than to remove the root and subject the patient to the necessity of wearing a plate. While it is possible and practicable to save the root of a tooth, it should be allowed to remain, and perform the function for which it was designed. The pathological condition of the teeth and oral cavity generally should receive careful attention at the hands of the dentist. From childhood to old age there are many diseases of the teeth, gums and alveoli that require attentive care, or serious results are sure to supervene. But it is impossible, nor is it necessary, to allude to them at this time, as they will be treated, or have been, in papers devoted to those special points.

Each year is developing some new thought, new method, new material. May it be ours to discriminate in their application, and act intelligently to elevate the standard and advance the interests of our profession.—*Report of Illinois State Dental Society.*

